# A cis-eQTL allele regulating reduced expression of *CHI3L1* is associated with late-onset adult asthma in Japanese cohorts

**DOI:** 10.1186/s12881-019-0786-y

**Published:** 2019-04-02

**Authors:** Jun Kanazawa, Haruna Kitazawa, Hironori Masuko, Yohei Yatagai, Tohru Sakamoto, Yoshiko Kaneko, Hiroaki Iijima, Takashi Naito, Takefumi Saito, Emiko Noguchi, Satoshi Konno, Masaharu Nishimura, Tomomitsu Hirota, Mayumi Tamari, Nobuyuki Hizawa

**Affiliations:** 1 Department of Pulmonary Medicine, University of Tsukuba, Tennodai 1-1-1, Tsukuba, Ibaraki, 305-8575 Japan; 2 Tsukuba Medical Center, Amakubo 1-3-1, Tsukuba, Ibaraki, 305-8558 Japan; 3 National Hospital Organization Ibaraki Higashi National Hospital, Terunuma 825, Tokai, Ibaraki, 319-1113 Japan; 4 Department of Medical Genetics, University of Tsukuba, Tennodai 1-1-1, Tsukuba, Ibaraki, 305-8575 Japan; 5 Department of Respiratory Medicine, Faculty of Medicine and Graduate School of Medicine, Hokkaido University, Kita15, Nishi7, Kita-Ku, Sapporo, Hokkaido 060-8638 Japan; 6 Research Center for Medical Science, The Jikei University School of Medicine, 3-25-8, Nishi-Shimbashi, Minato-ku, Tokyo, 105-8461 Japan

**Keywords:** Chitinase 3-like 1 (*CHI3L1*), YKL-40, Expression quantitative trait loci (eQTLs), Asthma, Genetics, Late-onset adult asthma

## Abstract

**Background:**

The chitinase-like protein YKL-40 plays a major role in inhibiting the inflammasome. Deregulation of inflammasome activation is emerging as a key modulator of pathologic airway inflammation in patients with asthma. We determined whether cis-expression quantitative trait loci (eQTLs) of the gene that encodes YKL-40, chitinase 3-like 1 (*CHI3L1*), are involved in the onset of asthma or in specific asthma phenotypes.

**Methods:**

This case-control study, which was conducted at the University of Tsukuba, Japan, included a total of 2709 adults from the Tsukuba genome-wide association study (GWAS) cohort (734 healthy volunteers and 237 asthma patients), the Tsukuba replication cohort (375 healthy adult volunteers and 381 adult asthma patients), and the Hokkaido replication cohort (554 healthy adult volunteers and 428 adult asthma patients). Among 34 cis-eQTLs in *CHI3L1* in the lung, rs946261 was associated with adult asthma in these Japanese cohorts. The genetic impact of rs946261 on asthma was also examined according to the age at onset and adult asthma clusters.

**Results:**

In the Tsukuba GWAS cohort, the C allele at rs946261 was significantly associated with reduced expression of *CHI3L1* mRNA in the lung and with development of asthma (odds ratio (OR) 1.27; *P* = 0.036). The association was also observed following analysis of the three Japanese cohorts (OR 1.16; *P* = 0.013). A stronger association was found with late-onset asthma that developed at 41 years of age or later (OR 1.24; 95% confidence interval (CI) 1.07–1.45; *P* = 0.0058) and with a specific asthma phenotype characterized by late onset, less atopy, and mild airflow obstruction (OR 1.29; 95% CI 1.03–1.61; *P* = 0.027).

**Conclusions:**

The genotype consisting of the cis-eQTL allele that reduces expression of *CHI3L1* was specifically associated with late-onset adult asthma. Given the important role of YKL-40 in many pathophysiological processes, including cell growth, migration, chemotaxis, reorganization, and tissue remodeling, it may be involved in an important pathogenic role in the establishment of inflammation and remodeling in asthmatic airways. Our findings may indicate the presence of a specific endotype related to exaggerated activation of YKL-40 in the pathogenesis of late-onset adult asthma.

## Background

YKL-40 is a prototypic mammalian chitinase-like protein. YKL-40 is expressed by multiple cell types in the lung, such as macrophages, neutrophils, and epithelial cells [1, 2]. This protein was increased in the serum from patients with community-acquired pneumonia severe enough to necessitate hospitalization [3]. Functionally, YKL-40 plays a crucial role in the regulation of response to bacterial infections by increasing the killing of bacteria, regulation of the correctness and strength of innate immune responses to bacteria, and facilitation of acquisition of adaptive immunity, healing, and repair [4].

Expression of YKL-40 is correlated with airway obstruction and assessment of airway remodeling including the thickness of the bronchial wall [5, 6]. Extracellular YKL-40 participates in inhibition of the inflammasome, followed by a decrease in the proinflammatory cytokine interleukin (IL)-1β [4]. However, the precise role of YKL-40 in asthma has not been clarified. Overactivation of the inflammasome is a new concept regarding modulation of pathologic airway inflammation in patients with asthma [7, 8]. Thus, we hypothesized that altered expression of YKL-40 may result in inappropriate inflammasome activation, and may thus play an important role in chronic pulmonary diseases such as asthma.

Chitinase 3-like 1 (*CHI3L1*), which is located on chromosome 1q31-q32 and includes 10 exons, encodes YKL-40 [9]. The rs4950928 polymorphism in *CHI3L1*, as shown in a genome-wide association study (GWAS), is associated with asthma risk, YKL-40 levels in blood, and respiratory function [10]. Other *CHI3L1* polymorphisms may play a role in development of asthma and are distinctly related to steroid-insensitive, non-T-helper cell type 2 chronic inflammation [11]. However, the results are inconsistent because of small sample sizes in previous studies, the presence of multiple causal variants at the loci, and differences in ethnicity, smoking exposure, and age. A systematic meta-analysis of five studies found no significant association [12].

Studying differences in gene expression can increase statistical power and improve interpretation of GWASs of susceptibility to complex diseases [13, 14]. Thus, we here examined the genetic impact of alleles associated with altered gene expression at expression quantitative trait loci (eQTLs) of *CHI3L1* on the development of asthma by performing a candidate gene case-control association study on 2709 adults from three independent Japanese populations, paying particular attention to the disease phenotypes.

## Methods

### Ethics statement

This study was approved by the Human Genome Analysis and Epidemiology Research Ethics Committee of the University of Tsukuba as well as by the Human Genome/Gene Analysis Research Ethics Review Committees of the Tsukuba Medical Center and of RIKEN. Written informed consent was obtained from each participant before the study, which was performed in accordance with the principles of the Declaration of Helsinki.

### Study participants

The Tsukuba GWAS cohort includes 971 Japanese individuals (734 healthy participants and 237 asthma patients) [15–17]. All individuals in this cohort underwent genome-wide single-nucleotide polymorphism (SNP) analysis [15]. To establish this cohort, healthy adults without respiratory diseases were recruited from among individuals who underwent an annual health exam at the Tsukuba Medical Center [18]. Patients with asthma, which was diagnosed by specialists in pulmonary medicine according to the criteria of the Japanese Society of Allergology [19], were recruited from the University of Tsukuba Hospital [20]. To eliminate the environmental effects of smoking in the present study, we excluded volunteers who smoked more than 10 pack-years. The multiple allergen simultaneous test-26 chemiluminescent assay was used to quantitate the levels of specific serum IgE antibodies [21]. Atopy was defined as a positive response (> 1.00 lumicount) to one or more of 14 common inhaled allergens.

Two independent Japanese cohorts, the Tsukuba and Hokkaido replication cohorts, were used to confirm the findings in the Tskuba GWAS cohort. Participants with a smoking history as described above were excluded. The Tsukuba replication cohort includes 375 healthy adult volunteers and 381 adults with asthma. The healthy adults in this cohort were also recruited from individuals who underwent an annual health exam at the Tsukuba Medical Center [18]. Individuals with asthma were recruited from the University of Tsukuba Hospital and underwent phenotypic and genetic analyses related to asthma [22]. The Hokkaido replication cohort includes 554 healthy adult volunteers and 428 adults with asthma who were recruited from Hokkaido University Hospital [15]. The original intent of studying these two cohorts was to determine genes that were associated with susceptibility to asthma in Japan.

### Search for eQTLs in CHI3L1

In complex diseases such as asthma, eQTLs play a significant role in trait associations in pathologically related tissues and explain a large proportion of heritability; expression of asthma-related genes is cis-regulated by asthma risk alleles in *TSLP*, *GSDMB*, *IL33*, *HLA-DQB1*, *C11orf30*, *DEXI*, *CDHR3*, *HCG22*, *HAS2*, and *ZBTB10* [16, 17, 23]. Thus, we first searched the GTEx Portal database (https://gtexportal.org/home/) for cis-eQTLs in *CHI3L1* in the lung that are located within 1 Mb of the transcription start site of *CHI3L1*.

### Genotyping

An automated DNA extraction system (QuickGene-610 L; Fujifilm, Tokyo, Japan) was used to isolate genomic DNA from whole blood samples. Samples from all individuals in the Tsukuba discovery cohort were used for GWAS genotyping, which was performed with Illumina HumanHap 550 k v3/610-Quad BeadChips (Illumina, San Diego, CA, USA) [15]. Rs946261 genotypes were determined in both replication cohorts using TaqMan allele-specific amplification (Applied Biosystems, Foster City, CA, USA).

### Statistical analysis

We initially used the Tsukuba GWAS data to examine the genetic effects of *CHI3L1* as one of the promising asthma candidate genes (ie, those previously associated in two or more non-Japanese cohorts) in Japanese populations. Also, given the ethnic difference in the LD pattern in the *CHI3L1* region, we tried a replication at the gene level but not at the SNP level. Six eQTLs that reside within 1 Mb of the transcription start site of *CHI3L1* were examined with logistic regression modeling for associations with asthma. As only rs946261 was associated with the presence of asthma in the Tsukuba GWAS cohort, this SNP was further genotyped in the Tsukuba and Hokkaido replication cohorts, and the combined results from the three independent cohorts were analyzed by adjusting for the cohort in the logistic regression model.

The age at onset of asthma was reported by each patient. To obtain the most accurate age at onset of asthma as possible, patients were questioned about dyspnea, wheezing, or coughing before the age of 18 years. When asthma onset was uncertain, the age at which the patient experienced the earliest respiratory symptoms was considered the age at onset of asthma symptoms [24].

To further examine the relationship between the age at onset of asthma and the genetic effects of the loss-of-function eQTL allele, we performed survival analyses of all the participants, with age at onset of asthma as the primary outcome. The Kaplan–Meier method was used to visualize the time required for development of asthma in subgroups stratified according to *CHI3L1* genotypes. This method was used to plot the proportion of the population that was asthma-free based on the individual’s age at the time of evaluation. A multivariate Cox proportional hazard regression model with the cohort and smoking index as covariates was used to estimate the hazard ratio (HR) of the effect of *CHI3L1* genotypes for the risk of asthma, which was reported with the 95% confidence interval (CI).

We also assessed the effect of the *CHI3L1* genotype using previously determined clusters of adult asthma classified by a multinomial logistic regression model to predict the probabilities of the different possible outcomes of specific phenotypes. In this previous study [22], we performed a two-step cluster analysis of 880 adult Japanese individuals with asthma and identified six phenotypes using eight variables: age, sex, age at disease onset, smoking status, total levels of serum IgE, percent of predicted forced expiratory volume in the first second (%FEV_1_), ratio of FEV_1_ to forced vital capacity (FVC), and specific IgE responsiveness to common inhaled allergens. The six phenotypes we identified were cluster A: older age at asthma onset, no airflow obstruction; cluster B: childhood asthma onset, no or mild airflow obstruction; cluster C: childhood asthma onset, long duration of asthma, and moderate or severe airflow obstruction; cluster D: older age at asthma onset, severe airflow obstruction; cluster E: intermediate age at asthma onset, no airflow obstruction; and cluster F: older age at onset, mild or moderate airflow obstruction. The two strongest variables for differentiation of these clusters were age at asthma onset and %FEV_1_. We then established a classification and regression tree (CART) model using these two variables [22]. In the current study, using this CART model, we assigned each adult patient with asthma to one of these clusters, with the result that 968 adult patients with asthma (574 in the Tsukuba cohort and 394 in the Hokkaido cohort) had the data for age at asthma onset and %FEV_1_, and were assigned to one of the six clusters. Among these 968 patients, 605 patients with asthma had been studied in the previous study [22], and the additional 363 patients with asthma were collected in the same geographic regions as the 605 patients. No differences in the clinical characteristics were found between the 605 and the 363 patients with adult asthma (data not shown). In addition, 1663 healthy adults (1109 in the Tsukuba cohort and 554 in the Hokkaido cohort) were included in the analysis as a seventh group and used as a reference in the multinomial model.

In the present study, considering that functional SNPs in the biological candidate may not yield significant *p*-values after correction for multiple testing but may replicate consistently over many populations tested, we used the liberal standard of “any nominal p < 0.05” to suggest a gene-level replication. To test whether the OR of the allele associated with altered gene expression for asthma was significantly greater or smaller than 1, two-sided *P* values less than 0.05 were considered significant. SPSS (version 22) was used for all analyses.

## Results

### Search for CHI3L1 eQTLs for a genetic association study

**Fig. 1:**
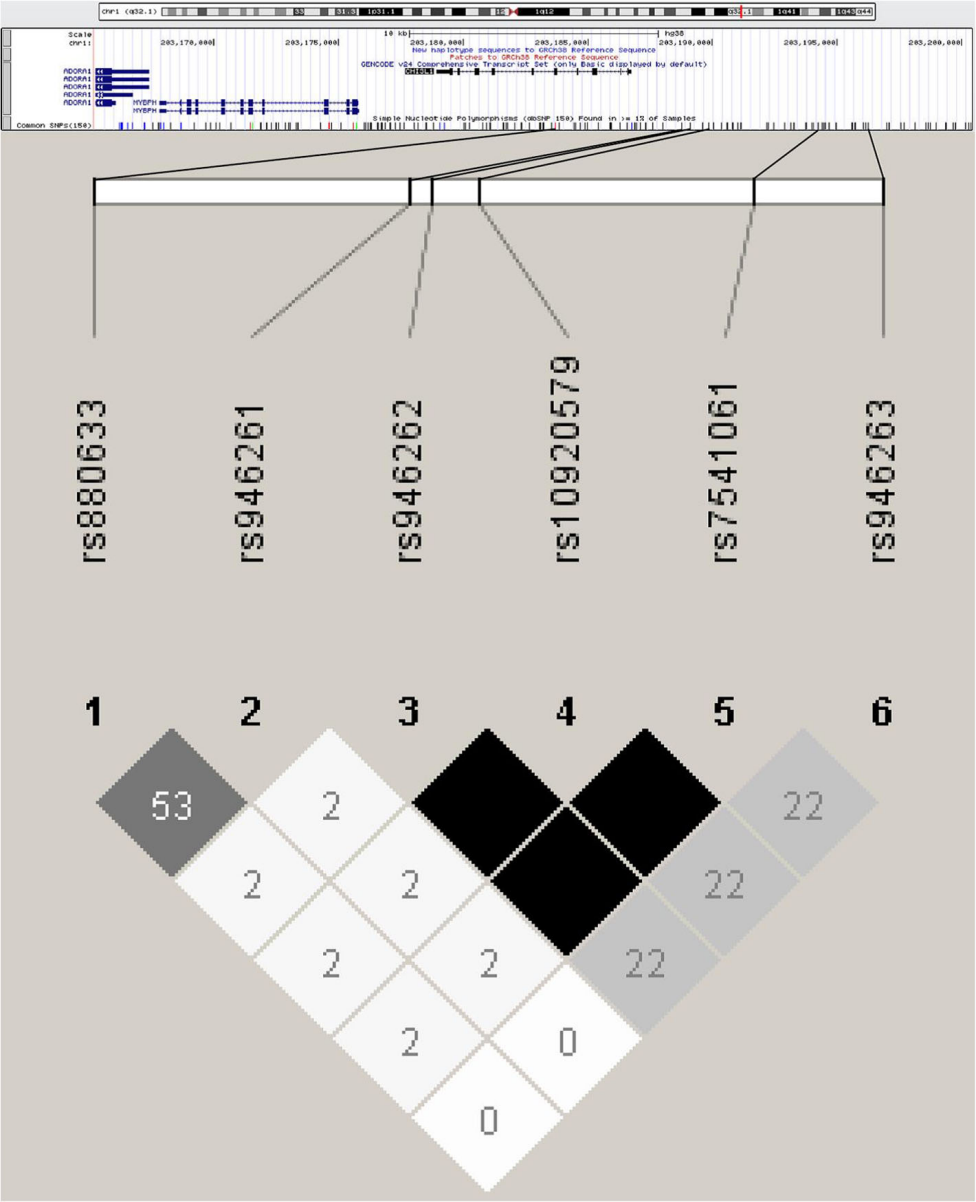
Pairwise LD (linkage disequilibrium) values (*r*^*2*^ × 100) for the six eQTLs of *CHI3L1* were calculated based on the genotypes of the Tsukuba GWAS cohort. The intensity of the gray shading in the squares of the LD map is proportional to *r*^*2*^

We identified 34 SNPs associated with expression levels of *CHI3L1* mRNA in the lung, six of which had previously been genotyped in the Tsukuba discovery GWAS cohort (Table 1; Fig. 1). The genotype frequencies of the three populations were in Hardy–Weinberg equilibrium.

**Table 1:** eQTL SNPs of *CHI3L1*

SNP	Chr1 position	eQTL	Minor allele	Risk allele	Minor allele frequency		Associations with asthma
		*P* value			HapMap JPT	Tsukuba GWAS cohort (Nonasthmatic + asthmatic patients)	*P* value
rs880633	203,183,673	2.5 × 10^−9^	C	T	0.31	0.36	0.30
rs946261	203,188,745	8.3 × 10^−9^	T	C	0.27	0.36	0.036
rs946262	203,189,101	9.9 × 10^−13^	T	T	0.052	0.048	0.94
rs10920579	203,189,844	2.6 × 10^−13^	A	A	0.052	0.048	0.94
rs7541061	203,194,239	6.3 × 10^−13^	A	A	0.052	0.048	0.94
rs946263	203,196,253	2.5 × 10^−11^	G	A	0.023	0.011	0.85

### rs946261 was significantly associated with asthma

The characteristics of the Tsukuba GWAS cohort, the Tsukuba replication cohort, and the Hokkaido cohort are shown in Table 2. Among the six eQTLs, the presence of the C allele at rs946261, which was significantly associated with reduced expression of *CHI3L1* mRNA in the lung (*P* = 8.30E-09), was associated with the development of asthma (OR 1.27; *P* = 0.036). We then examined this association in both the Tsukuba replication cohort and the Hokkaido cohort. The combined meta-analysis of the three independent populations showed that the presence of the loss-of-function C allele was associated with asthma (OR 1.16; *P* = 0.013) (Table 3). Rs946261 in the control groups of the three cohorts did not deviate from Hardy–Weinberg equilibrium (*P* > 0.5).

**Table 2:** Characteristics of the study population

	Tsukuba GWAS cohort		Tsukuba replication cohort		Hokkaido replication cohort	
	Nonasthmatic individuals	Asthmatic patients	Nonasthmatic individuals	Asthmatic patients	Nonasthmatic individuals	Asthmatic patients
Number of participants	734	237	375	381	554	428
Sex (female, %)	499 (68.0)	142 (59.9)	251 (66.9)	276 (72.4)	314 (56.7)	295 (68.9)
Age, y (range)	49.3 (27–74)	51.4 (20–75)	49.5 (22–78)	59.0 (19–100)	43.5 (18–84)	49.5 (16–83)
Age of asthma onset (range)	37.9 (0–70)			41.4 (1–88)		34.0 (0–80)
Smoking (%)						
Pack-year 0	607 (82.7)	196 (82.7)	311 (82.9)	313 (82.2)	492 (88.8)	331 (77.3)
0–10	127 (17.3)	41 (17.3)	64 (17.1)	68 (17.8)	62 (11.2)	97 (22.7)
Atopy (%)	412 (56.1)	151 (63.7)	257 (68.5)	175 (72.6)	304 (55.1)	300 (70.8)
FEV_1_% pred. (%, SD)	94.6 (12.3)	89.9 (20.1)	89.3 (14.1)	84.4 (23.2)	106.7 (14.5)	85.2 (23.8)
Serum IgE (log, SD)	1.68 (0.55)	2.22 (0.61)	1.90 (0.64)	2.18 (0.66)	1.80 (0.63)	2.27 (0.67)

Information on atopy, FEV_1_%pred., and serum IgE was missing in 27, 5, and 20 patients with asthma in the Tsukuba GWAS cohort, respectively

Information on FEV_1_%pred., was missing in 5 nonasthmatic controls in the Tsukuba replication cohort

Information on atopy, FEV_1_% pred., and serum IgE was missing in 140, 39, and 87 patients with asthma in the Tsukuba replication cohort, respectively

Information on atopy, FEV_1_% pred., and serum IgE was missing in 2, 170, and 11 nonasthmatic controls in the Hokkaido cohort, respectively

Information on atopy, FEV_1_% pred., and serum IgE was missing in 4, 48, and 4 patients with asthma in the Hokkaido cohort, respectively

**Table 3:** Results of association analysis in the three populations for rs946261

	Tsukuba GWAS cohort				Tsukuba replication cohort				Hokkaido replication cohort				Meta-analysis	
	n	RAF	OR	*P* value	n	RAF	OR	*P* value	n	RAF	OR	*P* value	OR	*P* value
		(C)	(95% CI)			(C)	(95% CI)			(C)	(95% CI)		(95% CI)	
Asthmatic patients	237	0.68	1.27	0.036	381	0.64	1.00	0.97	428	0.67	1.23	0.032	1.16	0.013
Nonasthmatic individuals	734	0.63	(1.02–1.59)		375	0.64	(0.81–1.23)		554	0.62	(1.02–1.48)		(1.03–1.31)	

*RAF* Risk allele frequency

### Association of rs946261 with specific phenotypes of asthma

**Fig. 2:**
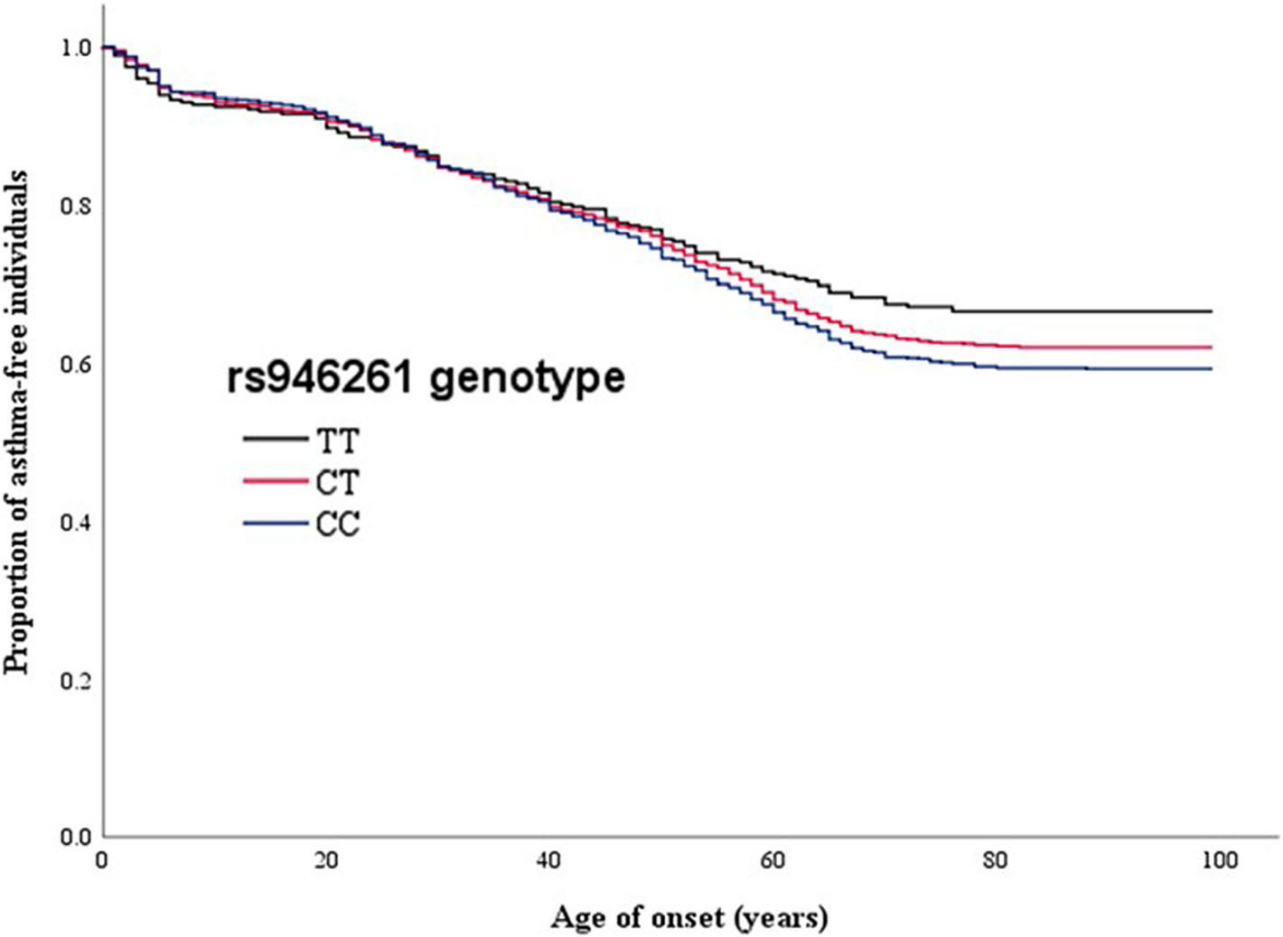
Kaplan–Meier curves for the proportions of asthma-free individuals. The *P* value for the association between genotype and the risk of asthma was 0.031 with multivariate Cox regression analysis

**Fig. 3:**
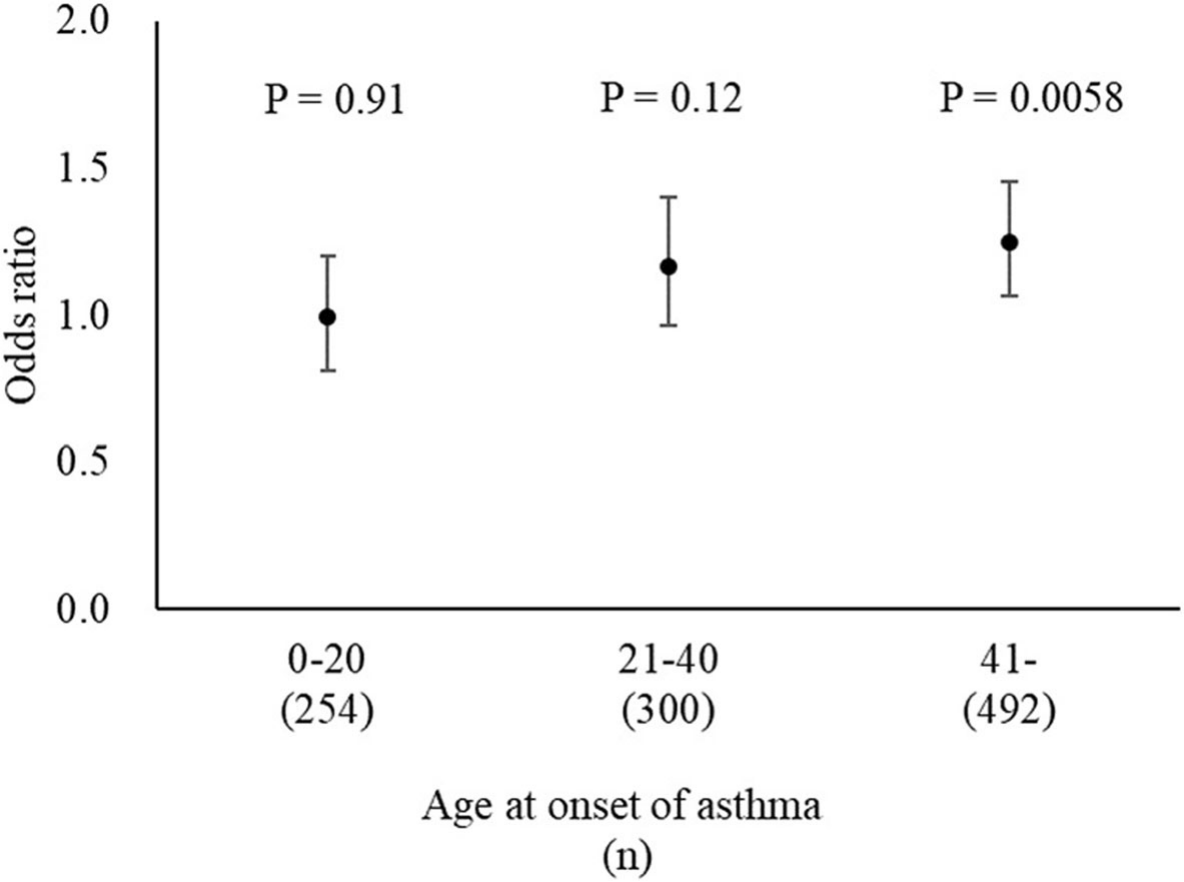
ORs for association between rs946261 and asthma in the three groups according to age at onset of the disease

Figure 2 shows Kaplan–Meier curves for the proportion of asthma-free individuals versus age of asthma onset for the three *CHI3L1* genotypes (CC, CT, and TT). The slope of the line for C carriers became steeper at approximately 40 years of age. Multivariate Cox regression analysis showed that the HR for the association between genotypes and risk of asthma was 1.11 (95% CI 1.01–1.21; *P* = 0.031). On the basis of the results of the Kaplan–Meier curve, we divided the patients with asthma into three groups according to age at onset of the disease (< 21 years, 21–40 years, and > 41 years). The *CHI3L1* genotype was significantly associated with asthma in patients who developed the disease at an older age (OR 1.24; 95% CI 1.07–1.45; *P* = 0.0058). On the other hand, we found no significant differences in the *CHI3L1* genotype distribution in patients with asthma who developed the disease at a younger age as compared with the healthy volunteers (Fig. 3).

We then used multinomial logistic regression analysis of each of the six clusters to look for an association between the *CHI3L1* genotype and specific asthma phenotypes. We found a significant association between the presence of the loss-of-function C allele and cluster A (OR 1.29; 95% CI 1.03–1.61; *P* = 0.027), which is characterized by late asthma onset, less atopy, and mild airflow obstruction (Table 4).

**Table 4:** Multinomial logistic regression between the *CHI3L1* SNP (rs946261) and the asthma clusters

	Phenotype	N	rs946261 RAF	*P* value	Odds ratio (95% CI)	Sex (Female, %)	Age (range)	Never smoker (%)	Age of asthma onset (SD)	FEV_1_% predicted (SD)	Atop (%)	Log IgE (SD)
Cluster A	Late-onset Mild Less-atopic	203	0.68	0.027	1.29 (1.03–1.61)	146 (72.3)	67.6 (50–88)	172 (84.6)	59.5 (9.4)	107.7 (12.8)	88 (56.4)	2.07 (0.62)
Cluster B	Early-onset Mild	200	0.63	0.88	0.98 (0.79–1.22)	112 (56.3)	35.4 (16–83)	159 (79.5)	10.3 (8.6)	89.3 (13.8)	162 (91.5)	2.48 (0.71)
Cluster C	Early-onset Moderate-to-severe	94	0.68	0.20	1.23 (0.90–1.69)	41 (44.1)	42.8 (19–82)	74 (78.7)	14.9 (10.0)	56.4 (11.6)	76 (88.4)	2.38 (0.59)
Cluster D	Late-onset Severe	91	0.66	0.41	1.14 (0.83–1.57)	66 (73.3)	65.2 (38–84)	75 (82.4)	48.6 (13.6)	49.5 (9.9)	47 (62.7)	2.22 (0.65)
Cluster E	Middle-age onset Female-dominant	155	0.67	0.14	1.21 (0.94–1.55)	127 (81.9)	46.8 (21–81)	111 (71.6)	34.7 (8.1)	104.3 (12.8)	95 (69.9)	2.04 (0.66)
Cluster F	Late-onset Moderate Less atopic	225	0.66	0.14	1.17 (0.95–1.44)	167 (74.2)	61.9 (31–88)	183 (81.3)	50.2 (12.1)	77.6 (8.7)	106 (57.9)	2.19 (0.67)

*RAF* Risk allele frequency

## Discussion

In the present study, we conducted a candidate gene case-control association study of asthma in three Japanese populations. We focused on *CHI3L1* because YKL-40 has been implicated in the pathogenesis of asthma [2, 5, 6], and [9] and shown to play a major functional role in inhibiting the inflammasome [3, 4], and [25]. In addition, we focused on functional SNPs and studied eQTLs [13, 14]. A meta-analysis of the three independent Japanese cohorts identified one late-onset asthma-associated eQTL, rs946261, in the 3′-untranslated region of *CHI3L1* (*P* = 0.0058). The risk allele rs946261[C] was also associated with a specific adult asthma cluster that is characterized by later onset, less atopy, and mild airflow obstruction. This risk allele for development of late-onset asthma was significantly correlated with reduced levels of *CHI3L1* expression. Given that YKL-40 acts as a negative regulator of the inflammasome and plays an important role in the process of inflammation resolution [25], dysregulation of resolution of inflammation caused by lower expression of YKL-40 in individuals carrying the risk allele may lead to increased susceptibility to development of late-onset asthma. Changes occur with age in both the innate and the adaptive immune responses, a phenomenon known as immunosenescence. Senescent cells do not proliferate but remain alive and show decreased or altered function. In the absence of infection, senescent cells show increased, low-grade, basal systemic inflammation (characterized by higher levels of IL-1β, IL-6, and tumor necrosis factor-α), which is known as inflammaging [26]. This inflammaging may partly explain why the genetic effect of *CHI3L1* was especially evident in asthma patients with disease onset at an older age.

Up to 40% of asthma patients older than 60 years experienced their first asthma symptoms after 40 years of age [27]. The underlying airway inflammation in asthma in adult-onset patients compared with that in childhood-onset patients is probably different and likely non-type-2 mediated, and is thus less likely to be successfully treated with standard-of-care asthma therapies [28]. Older asthma patients have more neutrophils in their sputum than do younger patients [29]. In a subgroup of patients with asthma and chronic obstructive pulmonary disease enrolled in a study called BIOAIR in Europe (Longitudinal Assessment of Clinical Course and Biomarkers in Severe Chronic Airway Disease), YKL-40 was identified as a steroid-insensitive, non-type-2 biomarker related to chronic inflammation [11]. In addition, an unsupervised cluster analysis of asthma patients previously identified subgroups according to the YKL-40 level, and the cluster with the highest serum YKL-40 levels was characterized by adult onset and less airflow obstruction. Airway transcriptome analysis in this cluster showed activation of non-type-2 inflammatory pathways [30]. The NLRP3 inflammasome (NLRP3: nucleotide-binding domain, leucine-rich-containing family, pyrin domain-containing-3 OR Nod-like receptor protein 3) is activated in response to a wide range of irritants including infectious agents, foreign particles, and endogenous factors involved in tissue injury and stress. Thus, this inflammasome may play a role in neutrophilic asthma, a subtype that is commonly induced by respiratory infections, smoking [31, 32], or obesity [33]. These previous studies together with the present study support the idea of an important role for dysregulated activation of the inflammasome in a specific phenotype of asthma characterized by later-onset, non-eosinophilic airway inflammation.

In contrast to the current study, the original association of *CHI3L1* with asthma was found with an increased *CHI3L1* mRNA expression. Some other studies also replicated the association between *CHI3L1* eQTL and asthma but with the risk allele reversed from the original report [34, 35]. The populations studied in the original report included one founder population consisting of genetically related individuals and other populations consisting of many children with asthma [10], which were totally different from the populations used in the replication studies consisting of mainly adult patients with asthma in outbred populations. In the current study, stronger association was found in adult asthma patients who developed the disease at 41 years of age or older, which may indicate that some *CHI3L1* SNPs have an opposite or independent effect on the risk of asthma later in life as compared with early-onset asthma.

We found no association between rs946261 and late-onset asthma in the Tsukuba replication cohort, and the genetic impact of the *CHI3L1* genotype on late-onset asthma was modest as a whole, with possible population heterogeneity. The current evidence suggests that the NLRP3 inflammasome and its related cytokines promote the development of allergic rhinitis [36, 37]. In addition, house dust mite-induced airway inflammation in mice significantly enhances the increased inflammasome activation triggered by rhinovirus infection [38]. Therefore, the larger number of atopic individuals in the Tsukuba replication cohort compared with the Tsukuba GWAS and Hokkaido replication cohorts may obscure the role of *CHI3L1* in the development of asthma in the Tsukuba replication population. A false-positive result may instead explain the modest association between *CHI3L1* and late-onset asthma. This false-positive result may be due to technical artifacts, heterogeneity in disease among patients, or an improper cut-off for significance in the statistical analysis. The efficiency and accuracy of our genotyping do not support the idea of technical problems. In addition, all three cohorts consisted of Japanese adults with smoking histories of no more than 10 pack-years. In any case, our finding cannot stand alone but should be seen as a contribution to the discussion about the genetic role of *CHI3L1* in asthma. Future studies are necessary and should involve sufficiently powered cohorts of asthma patients and subgrouping by disease phenotype to further understand how genetic variations in *CHI3L1* affect the risk for late-onset adult asthma.

## Conclusions

In conclusion, the loss-of-function *CHI3L1* genotype was specifically associated with late-onset asthma that developed at the age of 40 years or older. A previous study described different asthma endotypes, such as increased susceptibility to viral infections, increased bacterial colonization, abnormal lung development, and increased type-2 inflammation [39]. Given the important role of YKL-40 in many pathophysiologic processes, including cell growth, migration, chemotaxis, reorganization, and tissue remodeling, altered expression of YKL-40 may be involved in an important pathogenic role in the establishment of airway inflammation and remodeling, especially in specific phenotype(s) of asthma. Increased knowledge regarding the pathophysiologic role of YKL-40 in late-onset adult asthma will suggest new strategies for treating patients with specific endotypes early during the disease course, as specified by biomarkers or treatable characteristics of each endotype.

## Abbreviations

IgE: Immunoglobulin E
LD: Linkage disequilibrium
